# Investigation of the Oral Retention of Tea Catechins in Humans: An Exploratory Interventional Study

**DOI:** 10.3390/nu13093024

**Published:** 2021-08-29

**Authors:** Daisuke Furushima, Yu Otake, Natsumi Koike, Shintaro Onishi, Takuya Mori, Noriyasu Ota, Hiroshi Yamada

**Affiliations:** 1 Department of Drug Evaluation and Informatics, Graduate School of Pharmaceutical Sciences, University of Shizuoka, 52-1 Yada Suruga-ku Shizuoka, Shizuoka 422-8526, Japan; m16024@u-shizuoka-ken.ac.jp (Y.O.); hyamada@u-shizuoka-ken.ac.jp (H.Y.); 2 Biological Science Research Laboratories, Kao Corporation, Tokyo 131-8501, Japan; koike.natsumi@kao.com (N.K.); oonishi.shintarou@kao.com (S.O.); mori.takuya@kao.com (T.M.); ota.noriyasu@kao.com (N.O.)

**Keywords:** catechins, epigarocatechin-3-gallate, oral retention, xanthan gum, clinical study

## Abstract

Green tea catechin ingestion or gargling exhibit anti-viral activity against upper respiratory infection. We hypothesized that retention in the oral cavity could improve the anti-viral effects of catechins. The present study investigated the oral retention of catechins in humans and the effect of catechin beverage viscosity on oral retention. Two intervention studies with different test beverages, beverage-C (40 mL, containing 73.4 mg of catechins) and beverage-XT (40 mL, beverage-C containing 100 mg xanthan gum) were conducted in 20 healthy volunteers (mean age 38.7 years). Catechin concentrations were measured in buccal mucosa samples collected at 10 min, 40 min, and 60 min after ingesting test beverages, and the catechin variability of the tissue after intake was compared between test beverages. As a result, the mean (SEM) concentrations of EGCG were 99.9 (27.2), 58.2 (16.6), and 22.3 (5.7) ng/mg-mucosa at 10, 40, and 60 min, respectively, after ingestion of beverage-XT. Similarly, the catechin concentrations were 86.1 (20.3), 32.2 (5.3), and 27.8 (5.9) ng/mg-mucosa after ingestion of beverage-C. The total retention volume over 60 min tended to be slightly higher after ingestion of beverage-XT, though the difference was not statistically significant. Additional studies are needed to confirm the effect of xanthan gum on improving oral retention of catechins.

## 1. Introduction

Green tea, produced from the leaves of *Camellia sinensis* (L.) exhibits anti-tumor, anti-inflammatory, anti-microbial, and anti-viral activities, thereby conferring many health benefits to humans [1,2]. Catechins, classified as polyphenolic flavonoids, are the main active ingredient of green tea. Although the catechins in green tea leaves include many types of polyphenols, they contain especially high concentrations of epigallocatechin-3-gallate (EGCG), epicatechin 3-gallate (ECG), epigallocatechin (EGC), and epicatechin (EC). Among the catechins, EGCG is the major bioactive component, accounting for approximately 50% of the total catechin content in green tea leaves [3,4]. Although the mechanisms underlying the anti-viral effects of catechins are not fully understood, the galloyl (gallic acid ester) moiety at the 3-hydroxyl group of catechins might be an important contributor to anti-viral activity [5]. Various biological and pharmacological studies have revealed that the mechanisms of the anti-viral activity of EGCG against the influenza virus occurs via the inactivation of the viral envelope proteins or cellular receptors, or damage to the viral membrane, thereby blocking viral penetration into cell [5,6,7,8,9]. ECG, belonging to the galloyl group, also exhibits potent inhibitory effects on the growth of various influenza virus subtypes [5]. On the other hand, the anti-viral effects in humans have not been fully elucidated in clinical studies. Several randomized control studies have focused on the anti-viral effects against the influenza virus, but the results are inconsistent [10,11,12,13]. In particular, studies of tea catechin gargling (the movement of a liquid around in throat and oral cavity without swallowing) indicate a tendency toward a reduction in the incidence of influenza, but the results are neither statistically significant nor established with sufficient clinical evidence [14,15,16]. Catechin retention times in the oral cavity may be a cause of the divergence from such fundamental research. Therefore, it is important to clarify how long catechins are retained in the oral cavity after ingesting tea catechins, and whether retention in the oral cavity is a mechanism underlying the effects for controlling influenza infection in humans. The present study explored the retention of tea catechins in the human oral cavity, and investigated the effect of a high beverage viscosity on the retention of catechins in the oral cavity.

## 2. Materials and Methods

### 2.1. Subjects

Twenty healthy male volunteers working at Kao Corporation, aged 20 to 65 years, were recruited. The exclusion criteria included allergies or side effects to tea ingredients; undergoing treatment for hepatic, renal, or cardiac disease, respiratory disease, endocrine or metabolic disease, systemic immune disease, systemic infectious disease, or other diseases; trauma or inflammation in the oral cavity; an inability to stop taking foods or supplements containing tea catechins during the study period; and participation in a clinical trial other than this study. For all subjects, written informed consent was obtained prior to participation in the study. The study protocol was approved by the Ethics Committee of the University of Shizuoka (No. 1-2, allowed at 1 July 2019) and Kao Corporation (T211-190315, 23 April 2019). The outline of the study was registered as a clinical trial in the University Hospital Information Network (UMIN No. 000037474) before the start of the study.

### 2.2. Procedure

**Figure 1:**
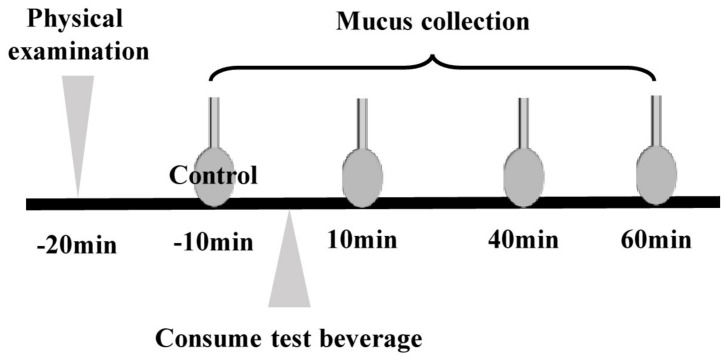
Outline of the trial. The test was carried out twice with different test beverages, beverage-C and beverage-XT. A wash-out period of at least one week was provided between the two trials.

The trials were performed from 22 August to 26 September 2019. All enrolled subjects participated in two interventional trials, consumption of a catechin-containing beverage (beverage-C) in the first trial and consumption of a catechin-containing beverage with xanthan gum (beverage-XT) in the second trial. Figure 1 shows the outline of the trial; all subjects were required to complete both sessions with the different test beverages. The two trials were performed under the same condition. In general, after participation was permitted by a medical doctor, participants were seated and asked to rest 10 min. Next, the oral mucosa of the inner cheek was collected and measured as a control value using a FLOQ swab (Copan Flock Technologies Srl, Brescia, Italy) after a physical examination by a medical doctor. The subject rested 10 min again, then consumed a 40 mL beverage (beverage-C or beverage-XT), and oral mucosa samples were collected with an FLOQ swab 10, 40, and 60 min after drinking. To collect mucosa from a defined area, a FLOQ swab was twisted five times while fixed to the mucosa inside the cheek. At least one week after the first trial, the second trial with the second test beverages was conducted with similar procedures. The locations of the mucus or mucosa collection at each time-point did not overlap. The amount of mucosa collected was calculated by subtracting the weight of the initial FLOQ swab from the weight of the FLOQ swab used to collect the mucus. The FLOQ swab with the collected mucosa was immersed in 200 μL of PBS containing 20 μL of 0.2% ascorbic acid, vortexed, and then the solution was dispensed into another tube and stored at −80 °C until the catechin content was determined. Food intake, strenuous exercise, smoking, alcohol intake, and health foods and supplements containing tea ingredients were prohibited after 21:00 the day before the trial.

### 2.3. Intervention Materials

Both of the beverages were manufactured by the Kao Corporation (Tokyo, Japan), and contained catechins, acidifiers, flavoring agents, vitamins, and sweeteners. Beverage-XT contained a total of 73.4 mg of catechins (including 27.9 mg of EGCG, 21.4 mg of EGC, 8.9 mg of ECG, 5.9 mg of EC, and 9.3 mg of other catechins) as well as 100 mg of xanthan gum, a neutral, water-soluble polysaccharide, which was included to increase the beverage viscosity. Beverage-C contained 69.7 mg catechins (including 26.1 mg of EGCG, 20.9 mg of EGC, 8.0 mg of ECG, 5.7 mg of EC, and 9.0 mg of other catechins), and no xanthan gum. The test beverages were manufactured in such a way to be indistinguishable from one another by taste, smell, and appearance. The test beverages, in powder form, were dissolved in 40 mL of water and the subjects drank the beverage immediately after mixing. The test beverage was consumed in three divided doses. The subjects were asked to ingest the test beverage after first holding it in the mouth and gargling for 3 s.

### 2.4. Determination of the Amount of Catechins in the Collected Mucosa

A total of 50 μL of MeOH, containing 10 μL of 0.2% ascorbic acid, was added to 50 μL of the thawed sample solution and vortexed. Then, 500 μL of ethyl acetate was added and the sample was vortexed for 4 min at room temperature and centrifuged at 2400× *g* for 5 min at 4 °C. Following this, 500 μL of the upper layer (organic layer) was collected in another 1.5 mL tube. To the remaining lower layer (aqueous layer), 500 μL of ethyl acetate was added again, and the same process described above was used to collect the upper layer. For the collected organic layer (a total of 1 mL), 10 μL of 0.2% ascorbic acid was added and the mixture was dried by centrifugation under reduced pressure. The obtained samples were then mixed with 100 μL of 10% acetonitrile solution containing 0.5% ascorbic acid, and the mixture was sonicated for 10 min. The amount of catechins present was analyzed by tandem mass spectrometry (MS/MS) detection.

### 2.5. Conditions for Tandem Mass Spectrometry Detection

An L-column2 ODS (2 μm, 3 mm × 50 mm; Chemicals Evaluation and Research Institute, Saitama, Japan), maintained at 40 °C, was used with a Nexcera system (Shimadzu, Tokyo, Japan), comprised of a degasser, feeding pump, autosampler, and column oven. The flow rate was 0.7 mL/min and the injection volume was 5 μL. The mobile phase was composed of 0.1% formic acid as eluent A and acetonitrile as eluent B. The initial elution solution was 10% B for 0.5 min, followed by a linear gradient to 15% B from 0 to 2.5 min, 27% B from 2.5 to 5.0 min, and 70% B from 5.0 to 5.01 min. This proportion was maintained for 1 min, and then the mobile phase was immediately returned to the initial condition and maintained for 1 min until the end of the run. Catechins were quantified by MS/MS detection in a positive ion mode using a 4500 Triple Quad system (AB SCIEX, Tokyo, Japan). The source/gas parameters for the collision gas, curtain gas, ion spray voltage, ion source gas 1, and ion source gas 2 were set at 8.0, 20.0, −4500, 60.0, and 70.0 L/min, respectively. The compound parameters, such as the declustering potential (DP), enhanced potential (EP), collision energy (CE), and collision exit potential (CXP), are shown Table 1. Quadrupoles Q1 and Q3 were set on unit resolution. Analytical data were processed using MultiQuant software (version 3.0.1).

### 2.6. Statistical Analysis

Descriptive statistics for all analyses are expressed as the mean and standard error of the mean (SEM) for continuous variables, and absolute and relative frequencies for nominal and ordinal variables. Catechin (EGCG, ECG, EGC, EC, and total catechin) variability, following ingestion of the two different test beverages, was compared by two-way repeated measures analyses of variance (ANOVA). Moreover, the area under the curve (AUC) and the maximum concentration (C_max_) of catechins were calculated from the distribution concentrations measured and compared between the test beverages using a Student’s t-test. A *p*-value of less than 0.05 was considered to indicate statistical significance. All statistical analyses were conducted using the statistical analysis program R (version 3.4.2, R Development Core Team 2018, R Foundation for Statistical Computing, Vienna, Austria).

## 3. Results

**Figure 2:**
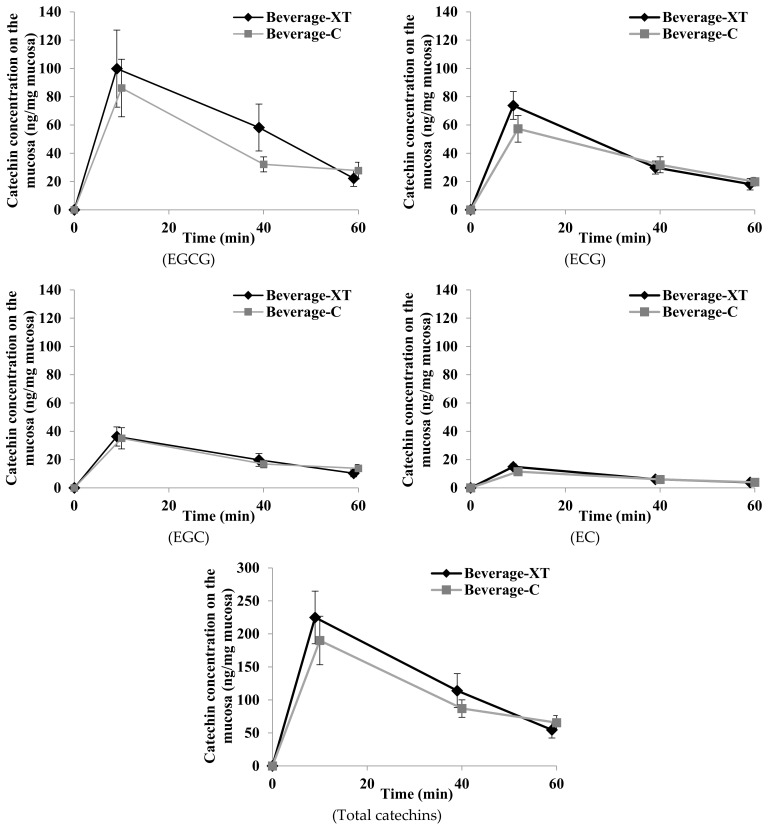
The mean (points) and standard error (error bars) of concentration of EGCG, ECG, EGC, EC, and total catechins at each specified time (10, 40, and 60 min); comparison between test beverages.

A total of 20 candidate participants were enrolled, 19 of whom fulfilled the study criteria; one volunteer was excluded due to stomatitis on the day of the trial. Subject age ranged from 26 to 62 years (with seven subjects in their twenties, six subjects in their thirties, two subjects in their forties, two subjects in their fifties, and two subjects in their sixties), with a mean (SD) age of 38.7 (11.7) years. Figure 2 shows the catechin concentration fluctuations following ingestion of beverage-C and beverage-XT. The concentration of each catechin evaluated gradually decayed from the peak immediately after administration, but catechins were still retained in the oral cavity at 60 min (Figure 2). At 10 min after ingestion of either beverage, the concentrations of EGCG, EGC, ECG, and EC were higher (in order) than the initial values before ingestion. The mean (SE) concentration (ng/mg-mucosa) EGCG, ECG, EGC, and EC at 10 min was 86.1 (20.3), 35.1 (7.5), 57.3 (9.4), and 11.5 (1.9), respectively, for beverage-C; and 99.9 (27.2), 36.3 (6.8), 73.8 (9.8), and 15.0 (1.9), respectively, for beverage-XT. Similarly, at 60 min, the mean (SEM) concentrations of EGCG, ECG, EGC, and EC were 27.8 (5.9), 13.8 (2.8), 19.9 (3.1), and 3.9 (0.6), respectively, for beverage-C, and 22.3 (5.7), 10.3 (2.5), 18.2 (4.1), and 3.9 (0.8), respectively, for beverage-XT. The catechin concentration fluctuations tended to be slightly higher after ingestion of beverage-XT than after beverage-C, but the difference did not reach statistical significance (*p* = 0.40 for EGCG, *p* = 0.96 for ECG, *p* = 0.47 for EGC, and *p* = 0.31 for EC). Table 2 shows the AUC values at 0 min to 60 min and the C_max_ of each catechin. As shown in Table 2, the AUC and C_max_ values tended to be slightly higher for beverage-XT than beverage-C, although the difference was not statistically significant.

## 4. Discussion

Several previous studies reported that green tea catechins, especially EGCG, have multifunctional bioactive molecules, which exhibit anti-bacterial and anti-viral activities. EGCG interferes with the interaction between virions and the host cells by its effects on the virion surface or cell surface receptors, and plays an important role in regulating the acidification of the endosomal and lysosomal microenvironment, which is crucial for viral invasion [5,6,7,8,9]. The clinical effectiveness of catechin ingestion, however, remains unknown. The present study investigated the oral retention of catechins after ingestion of a catechin-containing beverage in humans. The results indicated that a certain amount of catechins remained in the oral cavity even 60 min after ingestion of the catechin-containing beverage. The concentration of EGCG, in particular, was 86.1 (20.3) ng/mg at 10 min, and 27.8 (5.9) ng/mg at 60 min after ingestion of beverage-C, and 99.9 (27.2) ng/mg at 10 min, and 22.3 (5.7) ng/mg at 60 min after ingestion of beverage-XT. Assuming a specific gravity of saliva of 1.0 and a molecular weight of the EGCG compound of 458 g/mol, the value of the EGCG after 60 min can be converted to approximately 60.6 μM for beverage-C and 48.6 μM for beverage-XT. These EGCG levels in the oral cavity might represent an effective concentration for anti-viral activity in light of a previous study [5]. Furthermore, the findings of animal experiments using the solution with same catechin concentration indicated that the viral titer of the influenza virus was significantly reduced by an EGCG concentration of approximately 30 ng/mg-mucosa [17]. Moreover, it was recently reported that EGCG effectively inhibits enterovirus infection, even after brief exposure to the viruses and at various temperatures [18]. Thus, prolonged retention of catechins in the oral cavity might have a lasting anti-viral effect.

During the design phase of the study, we believed that increasing the viscosity of the catechin beverage would increase the retention of catechins in the oral cavity. Satisfactory evidence indicating that the addition of xanthan gum to the catechin-containing beverage enhanced catechin retention in the oral cavity was not obtained, although a slight tendency was detected. The catechin concentration varied greatly among the subjects, which may have prevented accurate analysis. In addition, the amount of xanthan gum might have been too small. As the test beverages contained catechin compounds with different hydrophilicities and molecular weights, factors that influence their penetration into the mucus layer, it is possible that the addition of xanthan gum affected their solubility. Basic research to evaluate this point further is required. Thus, the association between viscosity and the oral retention concentration of catechins cannot be confirmed, and further studies are necessary. Although additional studies are needed to clarify the conditions that enhance the anti-viral effects of catechins, the present study provides important data contributing to our knowledge of the anti-viral effects of catechins in humans. A previous randomized controlled study that investigated the preventive effects of the same green tea catechin beverage used in this study against acute upper respiratory infections reported that consumption of a beverage containing 171 mg of catechins per day for three months reduced the incidence risk of acute upper respiratory infections by 54% compared to the placebo beverage. The results of the present study contribute to the knowledge gained from these clinical trials and help to elucidate on methods for preventing acute upper respiratory infections or influenza in humans via catechins.

The present study has several limitations. First, in consideration of the safety of the subjects, mucosa was not collected in the pharynx, and instead, the buccal site was selected. As influenza viruses and other viruses that cause upper respiratory tract infections are known to mainly infect the pharynx, it is important to understand the concentration of catechins retained in this area. In addition, the collected oral mucosa swab might contain both mucosal epithelial cells and saliva. The analyzed data are therefore quite likely to represent catechins in the saliva as well as a fraction that may be present in the mucosal cells. To more accurately evaluate the oral retention of catechins, it is necessary to evaluate concentrations from various sites or in the saliva. Second, because the present study was conducted as an exploratory study in a specific, all male population with a small sample size, the generalizability of the obtained findings is limited. As such, the effect of xanthan gum on oral retention may be underpowered to demonstrate statistically significant differences between test beverages. Additional studies with a larger sample size, calculated on the basis of the present results, are needed. To the best of our knowledge, few studies have investigated the oral retention of catechins. Despite several limitations, the findings of present study might help to elucidate on the mechanisms of action of the anti-viral effects of catechins in the oral cavity. Recent studies reported an inhibitory effect of EGCG on COVID-19, which has increased attention toward green tea as a non-pharmacological preventive measure [19,20,21]. Although there are still many unknowns regarding the clinical effects of catechins in humans, we believe that the results of this study will guide future research related to catechins and contribute to their further development toward enhancing human health.

## 5. Conclusions

In summary, the present findings suggest that catechins, especially the galloyl group of catechins, which have anti-viral effects, remain in the oral cavity even 60 min after catechin ingestion. Adding xanthan gum to the beverage (beverage XT) slightly enhanced the retention of catechins, but the evidence was not sufficient. The next step in this research will be to investigate the possibility of prolonging oral retention by the addition of xanthan gum, and the relationship between the concentration of catechins in the oral cavity and clinical anti-viral effects.

## Figures and Tables

**Table 1:** Compound parameters used for quantification.

	Q1	Q3	DP	EP	CE	CXP
EGCG	457	169	−55	−4.5	−28	−4
ECG	441	169	−65	−4.5	−28	−4
EGC	305	125	−60	−4.5	−28	−2
EC	289	109	−55	−4.5	−34	−2

**Table 2:** Comparison of AUC_0–60_ (ng/mg-mucosa·min) and C_max_ (ng/mg-mucosa) of catechins.

Variables	Beverage-C	Beverage-XT	Difference	*p*-Value
EGCG				
AUC_0–60_	2805.3 (1939.0–3671.6)	3675.6 (2106.3–5244.8)	870.3	0.31
C_max_	98.1 (58.5–137.7)	123.7 (66.0–181.4)	25.6	0.45
ECG				
AUC _0–60_	1262.5 (909.0–1616.1)	1324.1 (913.4–1734.8)	61.6	0.81
C_max_	38.7 (24.0–53.4)	42.1 (28.1–56.0)	3.4	0.73
EGC				
AUC _0–60_	2142.7 (1606.6–2678.8)	2407.1 (1879.3–2934.9)	264.4	0.47
C_max_	62.3 (42.5–82.0)	77.9 (58.4–97.4)	15.7	0.24
EC				
AUC _0–60_	419.7 (316.2–523.3)	490.6 (387.2–593.9)	70.8	0.31
C_max_	12.1 (8.2–16.0)	16 (12.1–19.8)	3.8	0.15
Total catechins				
AUC _0–60_	6630.2 (4986.7–8273.8)	7897.3 (5562.5–10232.1)	1267.1	0.36
C_max_	7897.3 (5562.5–10232.1)	255.8 (172.5–339.2)	46.6	0.38

Data are expressed as mean (95% confidence interval); AUC_0–60_: area under the curve from 0 to 60 min after ingestion (unit ng/mg-mucosa·min); Cmax: peak catechin concentration; difference: difference between beverage-C and beverage-XT (beverage-XT-beverage-C); *p*-value (Wilcoxon rank sum test).
